# Faecal concentrations of ceftiofur metabolites in finisher pigs administered intramuscularly with ceftiofur

**DOI:** 10.1002/vms3.520

**Published:** 2021-05-15

**Authors:** Tara N. Gaire, Jessica Salas, Kara M. Dunmire, Chad B. Paulk, Mike D. Tokach, Tiruvoor G. Nagaraja, Victoriya V. Volkova

**Affiliations:** ^1^ Department of Diagnostic Medicine/Pathobiology Kansas State University Manhattan KS USA; ^2^ Department of Animal Sciences and Industry Kansas State University Manhattan KS USA; ^3^ Department of Grain Sciences and Industry Kansas State University Manhattan KS USA

**Keywords:** ceftiofur, dietary fibre, faeces, finisher pigs, metabolites

## Abstract

The objective of this study was to determine the effects of dietary fibre level and source on faecal ceftiofur metabolites concentrations after intramuscular administration of therapeutic ceftiofur hydrochloride in finisher pigs. Pens of finisher pigs (*n* = 36), with an equal number of barrows and gilts, were randomly assigned to 1 of 3 dietary treatment groups: basal diet composed of corn grain and soy bean meal with no supplement and formulated to contain 8.7% neutral detergent fibre (NDF), supplemented with 20% distillers dried grains with solubles (a byproduct of the ethanol production from corn grain) formulated to contain 13.6% NDF, primarily insoluble fibre or supplemented with 14.5% sugar beet pulp formulated to contain 13.6% NDF. Faecal samples were collected 6–8 hr after ceftiofur injection from treated and untreated pen‐mate pigs on days 1 and 3 of the 3‐day treatment regimen. Faecal concentrations of ceftiofur metabolites, including the major metabolite, desfuroylceftiofur, were analysed by reverse‐phase high pressure liquid chromatography with ultraviolet detection. Overall, the faecal concentrations of ceftiofur metabolites did not differ significantly between the dietary treatments. The mean concentrations of metabolites tended to be lower (*p* = .1) on day 3 compared to day 1 of the 3‐day treatment regimen. Faecal concentrations of metabolites were not affected by the gender of the finisher pigs. The concentrations of ceftiofur metabolites in the faeces are likely reflective of the microbial activity in the hindgut. Our data suggest that the fibre level and source used in the study did not affect the faecal concentrations of ceftiofur metabolites.

## Graphical abstract

The objective of this study was to determine the effects of dietary fiber level and source on fecal ceftiofur metabolites concentrations after intramuscular administration of therapeutic ceftiofur hydrochloride in finisher pigs. The concentrations of ceftiofur metabolites in the feces are likely reflective of the microbial activity in the hindgut. Overall, the fecal concentrations of ceftiofur metabolites did not differ significantly between the dietary treatments

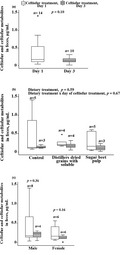

## 1 INTRODUCTION

Antimicrobials have been used in swine production, mainly in piglets and finisher pigs, to treat or prevent production‐related diseases as well as for growth promotion for more than 60 years (Cromwell, 2002; Lekagul et al., 2019). Ceftiofur, a third‐generation cephalosporin with a broad‐spectrum antimicrobial activity (NCBI, 2020), is commonly used in the USA to treat respiratory and other bacterial infections, such as enteritis, polyarthritis, and meningitis (Davies & Singer, 2020; Hornish & Kotarski, 2002). Ceftiofur is not commonly used outside the USA, particularly in the European Union, because of regulations associated with the use of third‐generation cephalosporins in food animals (More, 2020). Ceftiofur is used exclusively in animals, but is similar to ceftriaxone, another third‐generation cephalosporin, which is widely used in human medicine and is classified as a critically important antimicrobial by the World Health Organization (WHO, 2017).

The ceftiofur hydrochloride (Excenel^®^, Zoetis Animal Health) has been approved by the U.S. Food and Drug Administration for intramuscular injection in finisher swine. Following intramuscular administration, ceftiofur is rapidly metabolized to desfuroylceftiofur (DFC), a major metabolite (Beconi‐Barker et al., 1995). The DFC conjugates with macromolecules in plasma and tissues, or further metabolized to disulfides, such as DFC‐cysteine disulfide and DFC‐dimer (Beconi‐Barker et al., 1995; Beyer et al., 2015). The DFC and DFC dimer contain intact *β*‐lactam ring, therefore, are microbiologically active metabolites (Hornish & Kotarski, 2002). Most of the injected ceftiofur and its metabolites are excreted in the urine (60%–70%), but some (~10%–15%) is excreted in the faeces (Gilbertson et al., 1990). Urinary and faecal excretions of microbiologically active metabolites could contribute to the development and dissemination of antimicrobial resistance in bacterial populations in the pens and farm, resulting in resistant bacterial transmission to untreated animals. The entry of microbiologically active metabolites into the gut impacts the gut bacterial community structure and diversity (Foster et al., 2019: Ruczizka et al., 2019). Also, gut microbes are capable of producing enzymes that degrade ceftiofur and its metabolites to microbiologically inactive compounds, mainly by degrading the *β*‐lactam ring (Gilbertson et al., 1990). The extent of degradation of the ceftiofur active metabolites is likely to be affected by the diet because of its effects on the bacterial composition in the hindgut of swine. Among the dietary components, fibre is utilized during microbial fermentation in the hindgut, and is therefore likely to have a major impact on hindgut microbial composition (Metzler & Mosenthin, 2008). This study aimed to evaluate the effects of dietary fibre level and source on the concentration of ceftiofur metabolites in faeces after intramuscular injection of ceftiofur hydrochloride in finisher pigs.

## 2 MATERIALS AND METHODS

### 2.1 Animals, diets, and faecal sample collection and analysis

The animal use and procedures were approved by the Institutional Animal Care and Use Committee (IACUC). A total of 287 pigs (initial body weight 50.1 kg) were randomly distributed into 36 pens with seven or eight pigs per pen and aiming for an equal number of barrows and gilts in each pen. Pens were randomly assigned to one of three dietary treatments. The basal diet was corn grain and soybean meal‐based, and the three treatment groups were control diet with no supplement formulated to contain 8.7% neutral detergent fibre (NDF), supplemented with 20% distillers dried grains with solubles (DDGS) formulated to contain 13.6% NDF, or supplemented with 14.5% sugar beet pulp formulated to contain 13.6% NDF. The chemical compositions of the three diets are detailed in Table 1. The DDGS is a co‐product of ethanol production from grain and is commonly used in swine diets in the USA as a source of energy and protein (Stein & Shurson, 2009). Similarly, sugar beet pulp, an energy‐dense feed supplement rich in pectins and glucans, is a co‐product of sugar processing (Wang et al., 2016). The diets were fed to the finisher pigs in three phases: phase 1 from days 0 to 18, phase 2 from days 19 to 39, and phase 3 from days 40 to 86 (Table 1).

**TABLE 1:** Chemical analysis of diets fed to finisher pigs (as‐fed basis)^a^

Item	Phase 1 (days 0 to 18)			Phase 2 (days 19 to 39)			Phase 3 (days 40 to 86)		
	Control^1^	DDGS^2^	SBP^3^	Control^1^	DDGS^2^	SBP^3^	Control^1^	DDGS^2^	SBP^3^
Dry matter	88.9	89.8	89.9	89.2	90.4	90.4	88.4	89.4	89.0
Crude protein	17.4	18.7	16.7	14.3	16.4	14.3	12.6	15.3	12.3
Crude fat	2.6	4.0	5.6	2.9	4.5	5.8	2.9	4.0	4.8
Acid detergent fibre	3.0	3.8	7.3	3.5	4.4	6.8	2.5	3.9	7.6
Neutral detergent fibre	6.8	10.6	11.1	7.8	12.6	11.3	6.7	9.6	10.4
Nitrogen free extract	63.7	60.2	59.0	66.7	62.4	61.4	68.0	63.9	63.1
Ash	3.8	4.4	4.8	3.5	4.2	5.1	3.4	3.7	4.4

Note

The basal diet was corn grain (75% to 85%) and soybean meal.

^1^ Control: Basal diet formulated to contain 8.7% neutral detergent fibre (NDF).

^2^ DDGS: Basal diet supplemented with 20% distillers dried grains with solubles (DDGS), formulated to contain 13.6% NDF.

^3^ SBP: Basal diet supplemented with 14.5% sugar beet pulp, formulated to contain 13.6% NDF.

^a^ Analysis was performed by Ward Laboratories, Inc., on pooled feed samples.

Pigs that exhibited clinical signs of respiratory infection, diarrhoea, arthritis, or other production‐related diseases indicative of bacterial infections were treated with ceftiofur hydrochloride (Excenel^®^ RTU EZ, Zoetis Animal Health; 50 mg/ml) by intramuscular injection at 4.4 mg/kg body weight (4 ml/45.4 kg BW of the 50 mg/ml product) once a day for three consecutive days. On days 1 and 3 of the treatment regimen, faecal samples (~50 g into a Whirl‐Pak^®^ bag, Nasco) were collected from the animals per rectum approximately 6–8 hr after the ceftiofur injection.

The faecal samples were transported on ice to the laboratory, where well‐mixed sample aliquots were transferred to Eppendorf^TM^ centrifuge tubes (Thermo Fisher Scientific) and centrifuged for 15 min at 15,000 rpm. The supernatant was pipetted into a 2 ml‐0.45 µm filter Spin‐X^®^ centrifuge cryotube (Thermo Fisher Scientific) and again centrifuged at 5,000 rpm for 5 min. Faecal supernatants were stored at −80°C and the concentrations of ceftiofur metabolites in the faecal samples were analysed by reverse‐phase high pressure liquid chromatography with ultraviolet detection as described by Foster et al. (2016). In the assay, ceftiofur and desfuroylceftiofur conjugates are converted to a single stable derivative, desufuroylceftiofur acetamide, which is measured.

### 2.2 Statistical analysis

Descriptive statistics were obtained for the total ceftiofur and metabolite concentration of each day of the treatment regimen, dietary treatment, and gender. Statistical significance of the day of the treatment regimen and dietary treatment were tested using ANOVA in R software, R Core Team (2019). We also calculated whether interactions were present between the concentration and day of treatment, dietary treatment type and gender effects. Because of the modest sample size of the study (Table 2), the effect was considered as statistically significant if the ANOVA *p* < .10.

**TABLE 2:** Numbers of finisher pigs sampled for analysis of faecal concentration of ceftiofur metabolites during a 3‐day treatment regimen after intramuscular injection of ceftiofur hydrochloride

Day of the 3‐day treatment regimen	Dietary treatments		
	Basal diet of corn grain and soybean meal	Basal diet supplemented with 20% dried distiller's grains with solubles	Basal diet supplemented with 14.5% sugar beet pulp
1	6	4	5
3	3	4	3

## 3 RESULTS

**FIGURE 1:**
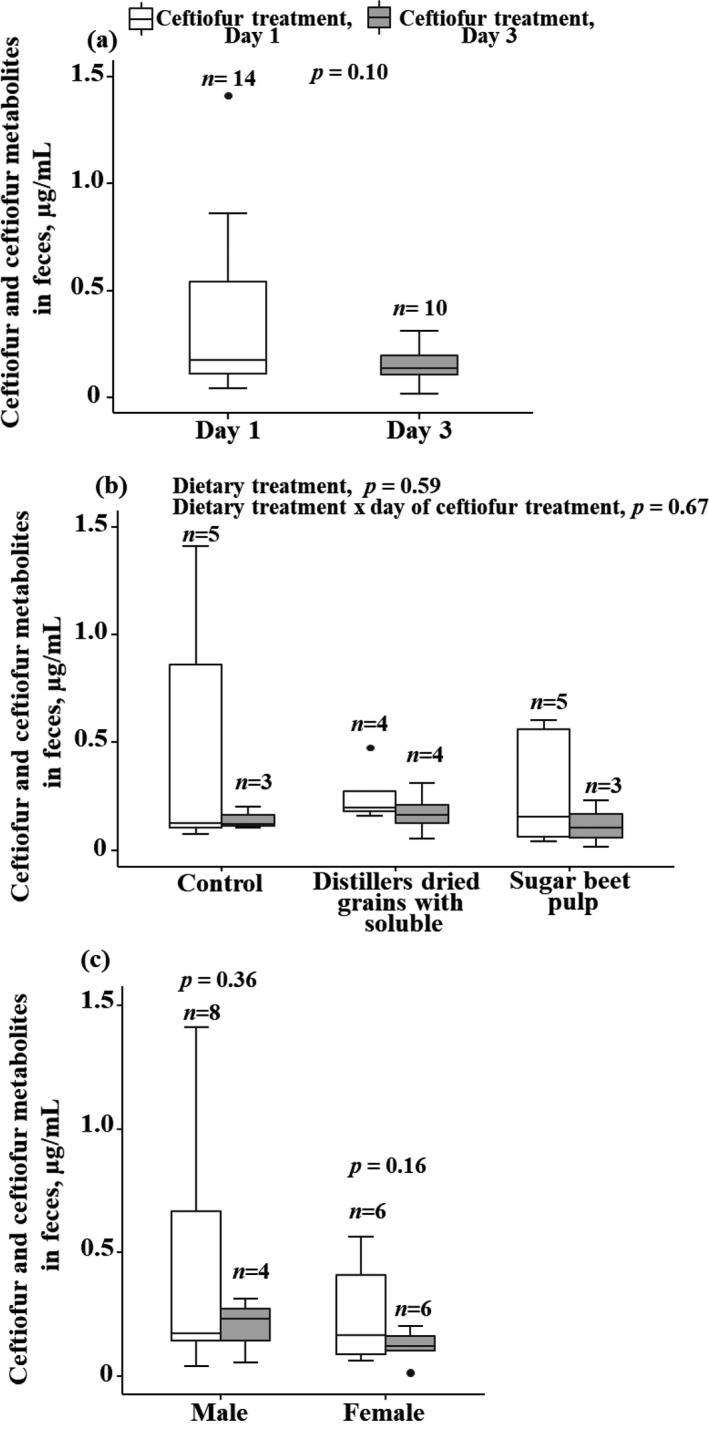
Boxplots of the total concentration of ceftiofur metabolites (µg/ml) in the feces of finisher pigs (a) on days 1 and 3 of the 3‐day treatment regimen of intramuscular injection of ceftiofur hydrochloride; (b) of the three dietary treatment groups, and (c) by gender. Ceftiofur hydrochloride was administered intramuscularly at 4.4 mg/kg body weight (4 ml/45.4 kg BW of the 50 mg/ml product) once a day for three consecutive days. Fecal samples were collected per rectum 6–8 hr post‐injection on days 1 and 3 of the treatment regimen. *n*, number of pigs. *p*‐values of ANOVA of statistical significance of the dietary treatment, animal gender, and day of the treatment regimen effects on the concentrations are shown

The measured NDF concentrations in the diets were lower than the formulated concentrations, but an increase in the fibre level in the second and third treatment groups compared to the control diet followed the expectations (Table 1). None of the pen mate control faecal samples had detectable concentrations of ceftiofur metabolites. Some of the pigs treated with ceftiofur on days 1 and 2 of the 3‐day treatment regimen showed a marked clinical improvement and were not injected with ceftiofur on day 3; faecal samples were not collected on day 3 from those pigs. The number of pigs sampled on days 1 and 3 of the treatment regimen are shown Table 2. The overall mean concentration of ceftiofur metabolites in pig faeces on day 3 of the treatment regimen (0.15 ± 0.08 µg/ml, *n* = 10) was lower (*p* = .1) than the concentration on day 1 (0.36 ± 0.39 µg/ml, *n* = 14) (Figure 1a). The mean concentrations of ceftiofur metabolites did not differ significantly between the three dietary treatments, and there was no significant interaction between the diet and day of the treatment regimen (Figure 1b). Also, the animal gender did not significantly affect the concentration, and there was no significant interaction between the gender and dietary treatments on the faecal concentration (Figure 1c).

## 4 DISCUSSION

We measured the concentrations of ceftiofur metabolites in faeces of finisher pigs that were treated with ceftiofur hydrochloride by intramuscular injection for 3 days for production‐related diseases and fed one of three diets differing in fibre level and source. The faecal samples were collected 6–8 hr post injection, which corresponds to the peak faecal concentrations of ceftiofur metabolites (Volkova et al., 2017). The diets were based on corn grain and soybean‐meal and supplemented with dried distiller's grains with solubles, a byproduct of ethanol production from corn grain, or with sugar beet pulp, a byproduct of sugar beet processing to achieve a higher level of NDF. The chemical and physical characteristics of dietary fibre influence the rate and extent of fibre degradation by the microbes in the hindgut (Jha & Berrocoso, 2015; Jha et al., 2019). Fibre in DDGS is primarily insoluble, hence, less degradable compared to fibre in sugar beet, which is soluble and more degradable (Li et al., 2020; Zhang et al., 2013). Fibre is a structural carbohydrate polymer that is indigestible in the stomach and small intestine and is microbially fermented in the large intestine of pigs. There are a number of studies that have shown that dietary fibre level and source have effects on microbial community, which include beneficial changes and improved gut barrier function (Heinritz et al., 2016; Le Sciellour et al., 2018; Shang et al., 2019; Wu et al., 2018). However, large intestinal microbial changes in relation to fibre level and type were not assessed in this study.

Because of its broad‐spectrum of activity, ceftiofur is a widely used antimicrobial for treating respiratory and other bacterial infections of swine in the USA. However, use of antibiotics in a therapeutic setting can cause selective pressure that may drive the development of antimicrobial resistance (AMR) and risk of a foodborne route of AMR from food animals to humans or the environment (Woolhouse et al., 2015). Thus, due to the growing concern over AMR, alternative approaches such as dietary interventions have been investigated.

The main finding of the study was that the mean concentrations of ceftiofur metabolites were lower in faeces of pigs on day 3 of the 3‐day treatment regimen compared to day 1 (*p* = .10) across all the dietary treatment groups and irrespective of the animal gender (both *p* > .10). Furthermore, we observed that the concentration was more predictable (had a tighter distribution) on day 3 compared to day 1 across dietary treatments (Figure 1a). Ceftiofur's *β*‐lactam ring is crucial for its antibacterial activity, but it is susceptible to hydrolytic degradation by *β*‐lactamases secreted by enteric bacteria (Dolhan et al., 2014). There is evidence that *β*‐lactamase production is dose‐dependent, increasing in response to increased flow of *β*‐lactam (Livermore, 1995). Earlier studies have demonstrated that ceftiofur metabolite degradation in animal faeces is biotic (Gilbertson et al., 1990), and thus the intestinal microbial community is contributing to the degradation of the *β*‐lactam ring of the metabolites (Beyer et al., 2015; Gilbertson et al., 1990; Hornish & Kotarski, 2002; Stentz et al., 2014). Further, several studies (Erickson et al., 2014; Rafii et al., 2009; Wagner et al., 2011) have isolated and characterized diverse bacteria (*Bacteroides* and *Bacillus*, etc.) from bovine faeces and gut contents that are capable of degrading ceftiofur. *Bacteroides*, a dominant member of the gut microbiome, may be contributing a large fraction of *β*‐lactamases, thus protecting other microbiome members from *β*‐lactams that reach the gut (Stiefel et al., 2015). Moreover, the *β*‐lactamase production can be expected to be dose‐dependent, accelerating in response to an increase in the *β*‐lactam concentration (Livermore, 1995) in the gut. Thus, a decrease in the *β*‐lactam concentration in animal faeces from day 1 to day 3 of the treatment regimen may be due to the expansion of *β*‐lactamase production by the intestinal microbiome by day 3, compared to an indigenous *β*‐lactamase level present on day 1. Although faecal metabolite concentrations were not affected by the animal gender, there is evidence of gender‐related differences in the metabolism of ceftiofur, secretion of metabolites into the intestine and changes in bacterial diversity in the gut (Ruczizka et al., 2019). Therefore, further research on the identification and characterization of *β*‐lactamase‐producing bacterial species from faecal samples in relation to fibre level and type, in addition to measuring drug metabolites, is warranted.

## 5 CONCLUSION

The concentration of ceftiofur metabolites in finisher pig faeces was lower on day 3 compared to day 1 of the 3‐day intramuscular ceftiofur treatment and was not affected by the animal gender or the level and source of dietary fibre.

## ANIMAL WELFARE AND ETHICS STATEMENT

The animal use procedures were approved by the Institutional Animal Care and Use Committee (IACUC) of Kansas State University.

## DISCLAIMER

This study was conducted while Victoriya Volkova was employed at Kansas State University. The opinions expressed in this article are the author's own and do not reflect the view of the National Institutes of Health, the Department of Health and Human Services, or the United States government.

## CONFLICT OF INTEREST

Authors declare no conflict of interest.

## AUTHOR CONTRIBUTION

**Tara N Gaire:** Investigation; Writing‐original draft. **Jessica Salas:** Investigation; Writing‐review & editing. **Kara Dunmire:** Investigation. **Chad Paulk:** Resources. **Mike D. Tokach:** Resources. **Victoriya Volkova:** Conceptualization; Writing‐review & editing. **TG Nagaraja:** Writing‐review & editing.

### PEER REVIEW

The peer review history for this article is available at https://publons.com/publon/10.1002/vms3.520.

## Data Availability

The dataset generated during this study is available from the corresponding author upon request.
